# The use of balloons for uterine cervical ripening is associated with an increased risk of umbilical cord prolapse: population based questionnaire survey in Japan

**DOI:** 10.1186/s12884-015-0432-4

**Published:** 2015-01-22

**Authors:** Junichi Hasegawa, Akihiko Sekizawa, Tomoaki Ikeda, Mitsuhiko Koresawa, Isamu Ishiwata, Masakiyo Kawabata, Katsuyuki Kinoshita, Group: Japan Association of Obstetricians and Gynecologists

**Affiliations:** Department of Obstetrics and Gynecology, Showa University School of Medicine, 1-5-8 Hatanodai, Shinagawa-ku, Tokyo 142-8666 Japan; Department of Obstetrics and Gynecology, Mie University School of Medicine, Mie, Japan; Kawakita General Hospital, Tokyo, Japan; Ishiwata Obstetrics and Gynecology Hospital, Ibaraki, Japan; Doai Memorial Hospital, Tokyo, Japan; Seijo-Kinoshita Hospital, Tokyo, Japan

**Keywords:** Cervical ripening balloon, Emergency cesarean section, Fore-lying cord, Perinatal mortality, Umbilical cord prolapse

## Abstract

**Background:**

To clarify whether the use of balloons for cervical ripening is associated with the incidence of umbilical cord prolapse.

**Methods:**

A postal questionnaire survey was distributed in Japan. Cases of umbilical cord prolapse occurring during labor in association with the use of balloons for cervical ripening between 2007 and 2011 in Japan were analyzed.

**Results:**

Answers from 942 institutions were obtained. The subjects included 369 patients with fore-lying or prolapse of the umbilical cord among a total of 2,037,460 deliveries. Among the singleton vertex cases, fore-lying or prolapse of the umbilical cord during labor were observed in 88 (0.005%) of 1,891,189 deliveries not associated with the use of balloons for cervical ripening and in 93 (0.064%) of 146,271 deliveries associated with the use of balloons for cervical ripening (Odds ratio 13.67, 95% confidence interval 10.21, 18.30). All types of balloons were significantly associated with the occurrence of fore-lying or prolapse of the umbilical cord. A total of 39% of cases of umbilical cord prolapse occurred during manual or spontaneous balloon removal, while 53% of cases occurred after a while not directly associated with balloon removal.

**Conclusion:**

The risk of umbilical cord prolapse was significantly increased during the use of balloons for cervical ripening, especially in cases involving the use of disk-type and ball-type balloons filled with large amounts of water.

## Background

Umbilical cord prolapse can result in poor neonatal outcomes because it may cause the cord to be compressed between the fetus and the maternal bony pelvis or soft tissue, inducing fetal hypoxia [1]. It is previously reported that incidence of umbilical cord prolapse ranges from 0.1 to 0.6% [2-6]. Although the total perinatal mortality and morbidity rates have been decreasing in Japan in association with improvements in neonatal resuscitation and newborn care, umbilical cord abnormalities including umbilical cord prolapse are still remaining causes of unfavorable perinatal outcomes, because cord prolapse can quickly lead to fetal compromise, with resultant long-term disability or death [1,7-10].

Several risk factors associated with umbilical cord prolapse, including fetal anomaly, fetal malpresentation, multiple pregnancy, polyhydramnios, preterm delivery, a birth weight less than 2500 g, preterm premature rupture of membranes [1,2,7,11,12]. Iatrogenic risk factors for umbilical cord prolapse also have been previously reported. Such factors are related to interventions that cause the fetal presenting part to be elevated out of the pelvis or occur following the rupture of the amniotic sac [1]. These interventions include artificial rupture of the membranes, attempted rotation of the fetal head, amnioinfusion, external cephalic procedures in a patient with ruptured membranes, placement of an intrauterine pressure catheter or fetal scalp electrode and the use of cervical ripening balloon catheters [1]. It has been reported that approximately 47% of cases of umbilical cord prolapse can be attributed to iatrogenic factors [8,13].

In these iatrogenic factors, cervical ripening balloons are often used to induce labor in Japan. Although the occurrence of umbilical cord prolapse during the antenatal period is not preventable in most cases, we believe that it is necessary to clarify the relationship between the incidence of umbilical cord prolapse and the use of cervical balloons in order to reduce the morbidity and mortality associated with umbilical cord prolapse. Hence, the accumulation of evidence regarding the relationship between umbilical cord prolapse and the use of balloons for cervical ripening is needed.

Therefore, we conducted a population-based survey of cases of umbilical cord prolapse collected from throughout Japan. The purpose of the present study was to clarify whether the use of balloons for cervical ripening is associated with the occurrence of umbilical cord prolapse.

## Methods

We conducted a postal questionnaire survey in Japan between August 2012 and June 2013 as an investigation of the Japan Association of Obstetricians and Gynecologists. A total of 2,683 institutions that provide maternity services across Japan were identified from a hospital list. Three pages of questionnaires regarding cases of umbilical cord prolapse and the total number of deliveries in each institution between 2007 and 2011 were sent to these hospitals.

The questions regarding umbilical cord prolapse after 22 weeks’ gestation included maternal characteristics and complications, timing of prolapse, use of a balloon for cervical ripening, fetal presentation, gestational age and timing of rupture of the membranes. Answers were based on respective medical records and databases which each hospital held. Each questionnaire was accompanied by a cover letter outlining the aims of the study and was addressed by name to the director, chief obstetrician or consultant in fetomaternal medicine. Answers to the questionnaires were received via facsimile.

**Figure 1:**
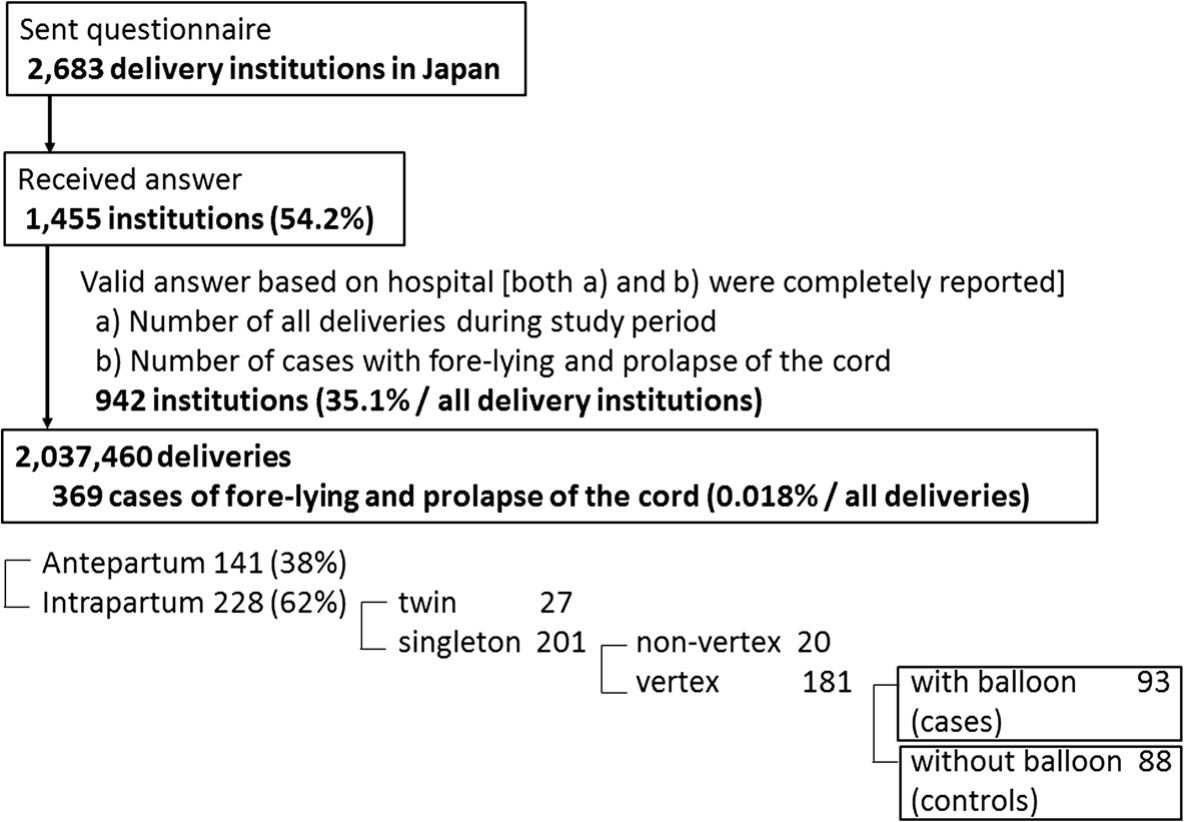
**Study flow diagram.**

Only fully completed answers regarding the number of cases with fore-lying or prolapse of the cord, the number of deliveries and the number of cases involving the use of balloons for cervical ripening during the study period were included in the present study. Among these cases involving fore-lying or prolapse of the cord during intrapartum, singleton vertex of the subjects were divided into cases which were associated with the use of balloons for cervical ripening and controls which were not associated with the use of balloons (Figure 1). The incidence of fore-lying or prolapse of the umbilical cord was then compared between the cases and the controls.

Umbilical cord prolapse was defined as a rupture of the fetal membranes and protrusion of the cord in advance of the fetal presenting part through the cervical os and into or beyond the vagina. Fore-lying of the umbilical cord was defined as the occurrence of an intact fetal membrane in cases in which the umbilical cord preceded the presenting part diagnosed using palpation through the membrane and/or transvaginal ultrasonography.

**Figure 2:**
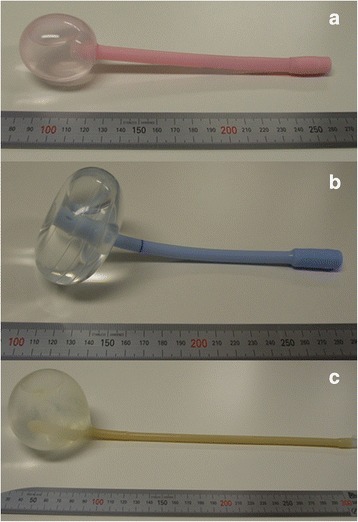
**Balloons for cervical ripening: (a) Intra-cervix balloon (usually filled with 40 ml of water and inserted into the uterine cervix), (b) Disk-type balloon (usually filled with 100 ml of water and placed into the uterine isthmus), (c) Ball-type balloon (usually filled with more than 100 ml of water and placed in the uterine isthmus).**

In most hospitals in Japan, the following three types of balloons are used for cervical ripening: (a) Intra-cervix balloons (usually filled with 40 ml of water and inserted into the uterine cervix), (b) Disk-type balloons (usually filled with 100 ml of water and placed into the uterine isthmus), (c) Ball-type balloons (usually filled with more than 100 ml of water and placed into the uterine isthmus). In cases involving the use of these balloons, the type and amount of water employed to inflate the balloon were recorded (Figure 2). Other types of balloons included double balloon catheter and gourd shape balloon.

### Statistical analysis

The frequency of fore-lying or prolapse of the umbilical cord was reported as the percentage and compared using Fisher’s exact test. Continuous variables were compared using Student’s *t*-test. Ordered variables were compared using the Mann–Whitney *U* test. Statistical significance was defined as a p-value of less than 0.05. The Statistical Package for Social Science (SPSS; Windows version 20.0 J; Chicago, IL, USA) was used for the analyses.

### Ethics statement

This study was performed as an investigation of the Japan Association of Obstetricians and Gynecologists (JAOG) and approved by the ethics board of JAOG. Because this was a retrospective analysis based on a questionnaire survey, patient information was anonymized and de-identified prior to answer to questions. Therefore, confidentiality of the patients involved was protected and no personal data were required for the present study.

## Results

We sent questionnaire to 2,683 delivery institutions in Japan and received replies from 1,455 (54.2%) institutions which had detail database associated with their delivery information. Following exclusion of answers with a deficient number of cases of fore-lying or prolapse of the cord and/or number of deliveries and cases involving the use of balloons for cervical ripening during the study period, answers from 942 institutions were collected in the present study. They included 369 patients with fore-lying or prolapse of the umbilical cord among a total of 2,037,460 deliveries.

A diagnosis of fore-lying or prolapse of the cord during intrapartum was made in 228 (62%) cases, while a diagnosis of them during antepartum period was made in 141. For final analysis, after exclusion of 27 twin pregnancies and 20 non-vertex presentations, a total of 181 singleton vertex cases with fore-lying or prolapse of the cord during labor were enrolled in the present study, and then 93 cases and 88 controls were analyzed. The demographics of these two groups did not differ except gestational weeks, as demonstrated in Table 1.

**Table 1:** **Demographics of the patients with fore-lying or prolapse of the umbilical cord among the singleton vertex cases**

	**Controls n = 88**	**Case used balloon n = 93**	**p-value**
***Maternal***			
Age	32.3 ± 5.0	31.9 ± 5.3	0.66
Gravida	0.5 (0–4)	0 (0-1)	0.49
Parity	1 (0–4)	0 (0–3)	0.50
Height (cm)	158.0 ± 5.8	156.8 ± 5.5	0.30
Weight at delivery (kg	63.2 ± 9.4	63.3 ± 10.3	0.98
***Neonatal***			
Gestational weeks	37.2 ± 4.6	38.9 ± 1.9	<0.01
Birth weight (g)	2677 ± 805	2677 ± 805	0.07
Apgar score 1 min.	6 (0–10)	8 (0-9)	0.50
5 min.	9 (0–10)	9 (0-10)	0.62
Umbilical artery pH	7.23 ± 0.15	7.21 ± 0.14	0.37
Base Excess	−7.0 ± 6.4	−6.4 ± 4.6	0.68
Intrauterine fetal death	4.5% (4)	2.2% (2)	0.43
***Other risk factor of umbilical cord prolapse***			
Low-lying placenta	0% (0)	0% (0)	1.00
Abnormal placental insertion	9.1% (8)	3.2% (3)	0.13
Polyhydroamnios	4.5% (4)	2.2% (2)	0.43
Oligohydramnios	0% (0)	2.2% (2)	0.50
***Diagnosis***			
Fore-lying	10.2% (9)	15.1% (14)	0.38
Prolapse			
*at spontaneous ROM*	29.5% (26)	40.9% (38)	0.16
*at amniotomy*	28.4% (25)	19.4% (18)	0.22
*after a while ROM*	31.8% (28)	24.7% (23)	0.32

The data indicate the mean ± standard deviation, median (range) or frequency (number of cases). ROM; rupture of membrane.

Balloons for cervical ripening were used in 146,271 cases (7.2% of all deliveries). The mean ± standard deviation of the amount of water used to inflate the balloon for cervical ripening was as follows: 43.0 ± 6.7 ml for intra-cervix balloons, 106.5 ± 9.3 ml for disk-type balloons and 130.9 ± 60.3 ml for ball-type balloons, respectively.

The incidence of fore-lying or prolapse of the umbilical cord in singleton vertex cases involving the use of balloons for cervical ripening is demonstrated in Table 2. Among the singleton vertex cases, fore-lying or prolapse of the umbilical cord during labor was observed in 88 (0.005%) of 1,891,189 deliveries not associated with the use of balloons for cervical ripening and in 93 (0.064%) of 146,271 deliveries associated with the use of balloons for cervical ripening (OR; Odds ratio 13.67, 95% CI; confidence interval 10.21, 18.30). All types of balloons were significantly associated with the occurrence of fore-lying or prolapse of the umbilical cord.

**Table 2:** **Incidence of fore-lying or prolapse of the umbilical cord associated with the use of balloons for cervical ripening during labor among the singleton vertex cases**

	**Incidence**	**Odds ratio (95% confidence interval)**
**Controls (without balloon)**	**0.005% (88/1891189)**	**Reference**
**When all cases regardless of the timing of the use of a balloon are calculated**		
**Total Cases**	**0.064% (93/146271)**	**13.67 (10.21, 18.30)**
Intra-cervix balloon	0.018% (10/56065)	3.83 (1.99, 7.37)
Disk shape balloon	0.060% (23/38348)	12.90 (8.15, 20.41)
Ball type balloon	0.120% (56/46640)	25.83 (18.48, 36.12)
Others	0.077% (4/5218)	16.49 (6.05, 44.92)
**When only cases directly associated with the use of a balloon are calculated (during use and at the time of removal)**		
**Total cases**	**0.030% (44/146271)**	**6.47 (4.50, 9.29)**
Intra-cervix balloon	0.004% (2/56065)	0.77 (0.19, 3.11)
Disk shape balloon	0.060% (14/38348)	7.85 (4.46, 13.80)
Ball type balloon	0.037% (28/46640)	12.91 (8.44, 19.75)
Others	0% (0/5218)	n/a

The incidence observed in the control group was calculated based on the total number of deliveries.

When only cases directly associated with the use of a balloon (during use and at the time of removal) were calculated, the incidence of fore-lying or prolapse of the umbilical cord was 0.030% (OR 6.47, 95% CI 4.50, 9.29). In this analysis, the frequency of fore-lying or prolapse of the cord did not differ between the cases in which intra-cervix balloons were used and the controls. Fore-lying or prolapse of the cord was diagnosed during the use of balloon in 3% (3/93) of the cases, during spontaneous balloon removal in 25% (23/93) of the cases and during manual balloon removal in 14% (13/93) of the cases, respectively. On the other hand, 53% (49/93) of the cases of umbilical cord prolapse occurred after a while (at least 15 min) not directly associated with balloon removal (not reported in 5% (5/93) of cases).

## Discussion

To our knowledge, this is the first large population-based investigation to demonstrate the exact prevalence of umbilical cord prolapse in association with the use of balloons for cervical ripening. In the present study, of all 369 cases, one-fourth of the present subjects (93 cases) experienced umbilical cord prolapse during and after the use of a balloon. The prevalence of fore-lying or prolapse of the umbilical cord was only 0.005% in cases not associated with the use of a balloon for cervical ripening, compared to 0.064% (OR 13.67) in the cases associated with the use of a balloon.

A previous study suggested that the use of a trans-cervical balloon catheter with 180–250 ml of saline increases the risk of cord presentation [14]. Similar to previous study, in particular, the use of a ball-type balloon filled with large amount of water (130.9 ± 60.3 ml) was associated with a remarkably high risk (OR 25.83). This odds ratio is highest among those of known risk factors in previous reports [3,4,7,12,15]. However, it is supposed that the increased risk of umbilical cord prolapse in cases involving the use of an intra-cervix balloon filled with approximately 40 ml of water was limited, as the incidence of umbilical cord prolapse after balloon removal did not differ between the patients treated with and without an intra-cervix balloon.

According to the answers to questions in which prolapse of the umbilical cord occurring during labor associated with the use of balloons for cervical ripening, umbilical cord prolapse occurred after a while balloon removal in more than half of the cases (53%). Even when umbilical cord prolapse did not occur during the use of a balloon or at removal, it may be possible to preserve the elevating fetal presenting part out of the pelvis and induce the wrong rotation of the fetal head, resulting in umbilical cord prolapse. Furthermore, the use of a balloon may involve occult umbilical cord prolapse during the procedure, after which umbilical cord prolapse is detected due to the identification of the descending fetal presenting part or rupture of the membranes. Unfortunately, only 57% of doctors participated in the present study answered that umbilical cord presentation was routinely confirmed using ultrasound scans during the use of a balloon for cervical ripening (data are not shown). Thus, the ultrasound confirmations of the umbilical cord presentation to diagnose fore-lying and occult prolapse of the umbilical cord before balloon placement, after and prior to removal might improve perinatal outcomes.

Questionnaire surveys in large population to obtain enough examples of a rare occurrence have limitations. Compared to western countries, there are a lot of small private hospitals that provide maternity services across Japan. Doctors worked such small hospitals did not retrospectively obtain detail obstetric information and did not answer to this questionnaire survey, because they were unlikely to have computerized database. Therefore, although we believe that the quality of obtained answers was good, this survey was limited by number of response.

Alternatively, since cases whose umbilical cord prolapse was found at just before delivery resulting in delivery without any neonatal complications might not be reported, prevalence of the umbilical cord prolapse might be underestimated. Besides, since the purpose of the present study was to clarify adverse effect of cervical balloon itself such as cord prolapse, the subjects were collected only singleton vertex cases complicated with fore-lying or prolapse of the cord during labor. Therefore, prevalence of fore-lying or prolapse of the cord in the present study was lower (0.005-0.064%) than in some former studies reported a prevalence of them ranging from 0.1 to 0.6% [2-6].

## Conclusion

Our findings revealed that the risk of umbilical cord prolapse was significantly increased during the use of balloons for cervical ripening, especially in cases involving the use of disk-type and ball-type balloons filled with large amounts of water.
