# Erratum to: The use of balloons for uterine cervical ripening is associated with an increased risk of umbilical cord prolapse: population based questionnaire survey in Japan

**DOI:** 10.1186/s12884-016-0948-2

**Published:** 2016-07-12

**Authors:** Junichi Hasegawa, Akihiko Sekizawa, Tomoaki Ikeda, Mitsuhiko Koresawa, Isamu Ishiwata, Masakiyo Kawabata, Katsuyuki Kinoshita, Group: Japan Association of Obstetricians and Gynecologists

**Affiliations:** Department of Obstetrics and Gynecology, Showa University School of Medicine, 1-5-8 Hatanodai, Shinagawa-ku, Tokyo, 142-8666 Japan; Department of Obstetrics and Gynecology, Mie University School of Medicine, Mie, Japan; Kawakita General Hospital, Tokyo, Japan; Ishiwata Obstetrics and Gynecology Hospital, Ibaraki, Japan; Doai Memorial Hospital, Tokyo, Japan; Seijo-Kinoshita Hospital, Tokyo, Japan

## Erratum

After publication of the original article [1], it came to the authors’ attention that a few values in Table 2 were added incorrectly. The correct values are as follows: for total cases ‘**disk shape balloon**’ and ‘**ball type balloon**’ the incidence observed was 0.037 % (14/38348) and 0.060 % (28/46640), respectively. All other values within Table 2 are correct.
